# *In Vitro* Cultivars of *Vaccinium corymbosum* L. (Ericaceae) are a Source of Antioxidant Phenolics

**DOI:** 10.3390/antiox4020281

**Published:** 2015-04-09

**Authors:** Rodrigo A. Contreras, Hans Köhler, Marisol Pizarro, Gustavo E. Zúñiga

**Affiliations:** Laboratorio de Fisiología y Biotecnología Vegetal, Departamento de Biología, Facultad de Química y Biología, Universidad de Santiago de Chile. L. B. O’Higgins Ave. #3363, Estación Central, Santiago of Chile, 9170022, Chile; E-Mails: hans.kohler@usach.cl (H.K.); marisol.pizarror@usach.cl (M.P.)

**Keywords:** high-bush blueberry *in vitro* cultivars, HPLC-DAD and LC-MS/MS, DPPH• and FRAP, phenolic composition

## Abstract

The antioxidant activity and phenolic composition of six *in vitro* cultured blueberry seedlings were determined. Extracts were prepared in 85% ethanol from 30 days old *in vitro* cultured plants and used to evaluate the antioxidant capacities that included Ferric reducing antioxidant power (FRAP) and 1,1-diphenyl-2-picrylhydrazin (DPPH•) scavenging ability, total polyphenols (TP) and the partial phenolic composition performed by high performance liquid chromatography with diode array detector (HPLC-DAD), liquid chromatography coupled to tandem mass spectrometry (LC-MS/MS (ESI-QqQ)). All ethanolic extracts from *in vitro* blueberry cultivars displayed antioxidant activity, with Legacy, Elliott and Bluegold cultivars being the most active. In addition, we observed a positive correlation between phenolic content and antioxidant activity. Our results suggest that the antioxidant activity of the extracts is related to the content of chlorogenic acid myricetin, syringic acid and rutin, and tissue culture of blueberry seedlings is a good tool to obtain antioxidant extracts with reproducible profile of compounds.

## 1. Introduction

Epidemiological studies have demonstrated that a diet rich in fruits and vegetables reduces the risk of certain types of cancer, cardiovascular, and other chronic diseases. Blueberries have been traditionally used for either fresh consumption or processed into jams, jellies and juices. Phenolic acids (benzoic acids and cinnamic acids), also known as non-flavonoids and their derivatives, are found in blueberries. The benzoic acids reported in blueberries are vanillic acid, syringic acid, gallic acid, protocatechuic acid, *m*-hydroxybenzoic acid, hydroxybenzoic acid and ellagic acid [1,2]. While the cinnamic acids found were chlorogenic acid, caffeic acid, ferulic acid, *p*-coumaric acid, *o*-coumaric, acid and *m-*coumaric acid [1]. Chlorogenic acid is the major phenolic present in the fruit [3]. It has been shown that chlorogenic acid can scavenge reactive oxygen species (ROS) and alkylperoxyl radicals, protecting macromolecules like deoxyribonucleic acid (DNA), proteins and membranes [4]. These properties convert chlorogenic acid into a good candidate to be used in the food industry, pharmacology and cosmetology. Then, the industrial production of antioxidant compounds from blueberries seems to be desirable. However, the supply of secondary metabolites has many limitations from fruits or leaves grown under field conditions such as biomass availability and vary in their chemical composition which is strongest affected by the environment [5]. There has been considerable interest in plant tissue culture as a potential alternative for the production of secondary metabolites [6]. The major advantages of a plant tissue culture system over the conventional cultivation of whole plants are as follows: the synthesis of bioactive secondary metabolites runs in a controlled environment, independent of climatic and soil conditions; negative biological influences that affect secondary metabolites production in the nature are eliminated (microorganisms and insects); and automated control of cell growth and rational regulation of metabolite processes would reduce labor costs and improve productivity [7].

In this work, we evaluated the use of *in vitro* culture of highbush blueberry cultivars of commercial importance in Chile as a continuous source of antioxidant compounds. In addition, we characterize the chemical composition of extracts by using HPLC-DAD and LC-MS/MS in order to produce standardized extracts.

## 2. Experimental Section

### 2.1. Chemicals

All solvents used were HPLC grade. Ethanol, methanol and acetonitrile were purchased from J.T Baker Chemical Co. (Phillipsburg, NJ, USA), phosphoric acid and hydrochloridric acid were purchased from Riedel-de-Haëhn (Seelze, Germany). Folin-Ciocalteu reagent, sodium carbonate anhydrous and formic acid were purchased from Merck Chemical Co. (Darmstadt, Germany), 1,1-diphenyl-2-picrylhidrazyl (DPPH•), 2,4,6-tris(2-pyridyl)-*s*-triazine (TPTZ), FeCl_3_·6H_2_O, sodium acetate and HPLC standards (gallic acid, quercetin, rutin, chlorogenic acid, caffeic acid, syringic acid, myricetin, *p*-coumaric acid, ellagic acid, kaempferol, naringenin, isoquercitrin, morin, genistein and luteolin) were purchased from Sigma-Aldrich Chemical Co. (St. Louis, MO, USA), Lloyd-McCown medium base and 6-γ,γ-dimethylallylaminopurine (2-iP) were purchased from Phytotechnology labs. (Kansas City, MO, USA), and finally acetic acid and agar-agar were purchased from Winkler Ltda. (Santiago, Chile).

### 2.2. Plant Material

*In vitro* cultures of *V. corymbosum cv.* Duke, Legacy, Brigitta, Elliott, Misty and Bluegold were started from shoot apices of pathogen free certified plants. Explants were cultured in a Lloyd-McCown medium base [8] supplemented with 2% of sucrose, 2.5 mg/L of 2-iP (cytokinin) and 7.5 g/L of agar-agar. Cultures were maintained during 30 days at 23 ± 2 °C with 16/8 light/darkness photoperiod.

### 2.3. Extracts Preparation

A total of 100 mg of fresh *in vitro* culture medium free shoots were mixed with 1 mL of ethanol (85% v/v) and sonicated at 50–60 Hz of frequency for two hours at 25 °C according the method previously described [9]. Extracts were filtered in a 0.45 μm pore filter (Millipore, Billerica, MA, USA).

### 2.4. DPPH• Free Radical-Scavenger Spectrophotometric Assay

The radical scavenging activity of the blueberry extracts was determined by using the 2,2-diphenyl-1-picrylhydrazyl radical (DPPH•) according the procedure previously described [10]. In its radical form, DPPH• has an absorption band at 517 nm, which disappears upon reduction by an antiradical compound. Briefly, 20 μL of each extract were added to 980 μL of daily-prepared DPPH• ethanolic solution (0.78 absorbance units). Absorbance at 517 nm was measured with an Agilent 8453 UV-Vis spectrophotometer (Palo Alto, CA, USA), 4 min after starting the reaction. Results were expressed as % of DPPH• consumed.

To determinate the 50% of inhibitory concentration (IC_50_), serial dilutions of extracts and/or phenolic standards were used to measure the scavenging of DPPH• radical as a function of serial dilution. Each determination was performed in triplicate and repeated at least three times and using a linear regression to calculate the concentration to scavenge the 50% of DPPH•.

### 2.5. Ferric Reducing/Antioxidant Power (FRAP)

The FRAP activity of the extracts was measured according to the method previously described [11]. Briefly, 5 μL of ethanolic extract was mixed with 900 μL of FRAP reagent and 95 μL of water (FRAP reagent was prepared to mix acetate buffer (300 mM, pH 3.6): TPTZ solution (10 mM in chloride acid) and ferric chloride solution (20 mM) (10:1:1 v/v). Absorbance at 593 nm was measured with an Agilent 8453 UV-Vis spectrophotometer, 4 min after starting the reaction. Results were expressed as ascorbic acid equivalents (AAE) per gram of dry weight (DW). Each determination was performed in triplicate and repeated at least three times.

### 2.6. Total Phenolic Content (TPC)

The total phenolic content was measured according to the method previously described [12]. 40 μL of the extract to be tested were added with 100 μL of Folin-Ciocalteu’s reagent, 560 μL of deionized water; reaction was stopped after 5 min at room temperature with 300 μL of 7% aqueous sodium carbonate. The absorbance was measured at 660 nm [13] on an Agilent 8453 UV-Vis spectrophotometer. The results were expressed in gallic acid equivalents (GAE) per gram of DW. Each determination was performed in triplicate and repeated at least three times.

### 2.7. Analysis of Extracts by HPLC-DAD

The determination of phenolic compounds was carried out using an Agilent high performance liquid chromatography system equipped with a UV-Vis photodiode-array detector (HPLC-DAD, 1100 series, Palo Alto, CA, USA). The chromatographic separation was obtained by a RP-C18 column (Zorbax, Eclipse XDB-C18 4.6 × 150 mm, 5 μm, Agilent Technologies, Inc., Santa Clara, CA, USA) with solvent A (acetonitrile) and B (1% phosphoric acid) under gradient conditions: 0 min, 10% of A, 5 min, 25% of A, 8 min, 35% of A, 15 min, 60% of A, 17 min, 35% of A and final 20 min, 10% of A. The flow rate was 1 mL min^−1^ and the column were thermostatically controlled at 25 °C. UV-detection was performed at 254, 280, 314 and 340 nm, the results were expressed as mg per gram of DW. The standards used were gallic acid, quercetin, rutin, chlorogenic acid, caffeic acid, syringic acid, myricetin, *p*-coumaric acid, ellagic acid, kaempferol, naringenin, isoquercitrin, morin, genistein and luteolin.

### 2.8. Analysis of Extracts by LC-MS/MS

The compounds in the extracts were analyzed by LC-MS/MS. LC-MS/MS was performed using an Agilent triple quadrupole mass spectrometer (MS/MS, 6400) equipped with an Agilent LC 1200 series. A RP-C18 column (Zorbax, Eclipse XDB-C18 4.6 × 150 mm, 5 μm) was used at flow rates of 1 mL min^−1^ at room temperature. Conditions for MS analysis include a capillary voltage of 4000 V, a nebulizing pressure of 40 psi, and the drying gas temperature of 330 °C. HPLC gradient was in acetonitrile (A) and 0.1% formic acid (B), as follows: 0 min, 10% of A, 5 min, 25% of A, 8 min, 35% of A, 15 min, 60% of A, 17 min, 35% of A and 20 min, 10% of A. Compounds were analyzed by both negative and positive ion mode.

### 2.9. Statistical Analysis

Statistical differences were determined using analysis of variance (ANOVA) with Tukey’s post-test for all samples. Significant differences were determined using 95% of confidence (*p* < 0.05). We use *n* ≥ 3 replicas in all experiments and measurements.

## 3. Results and Discussion

### 3.1. In Vitro Culture Establishment

**Figure 1:**
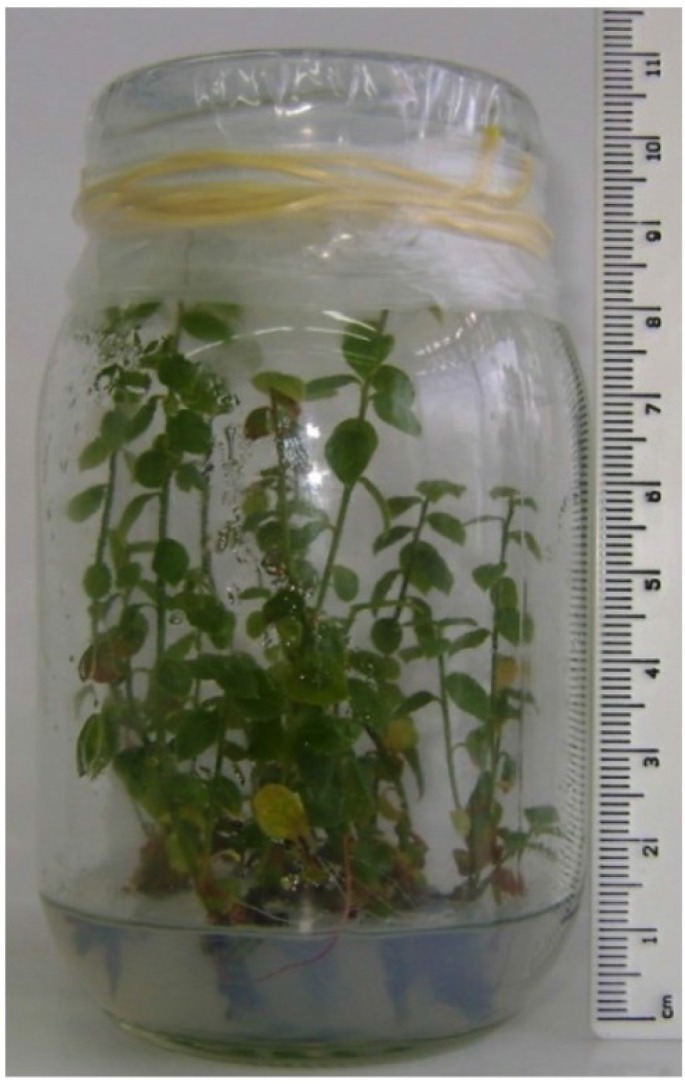
*In vitro* blueberry plants *cv.* Misty in supplemented Lloyd-McCown medium (see Experimental Section).

The six cultivars were successfully established using the McCown-Lloyd medium base [8] supplemented with 2-iP. The replication index of all cultivars was 8 after 30 days of culture. Figure 1 shows the appearance of blueberry *cv.* Misty. The other cultivars presented a very similar appearance. Shoots of one month were used to obtain the extracts used.

### 3.2. Antioxidant Activity of Extracts

Blueberry fruits are known to have a high content of polyphenols, e.g., anthocyanins, flavonols, isoflavonols, *etc.*, that are potent antioxidants and that may have protective effects against diseases related to free radical production, such as cancer and cardiovascular diseases.

**Figure 2:**
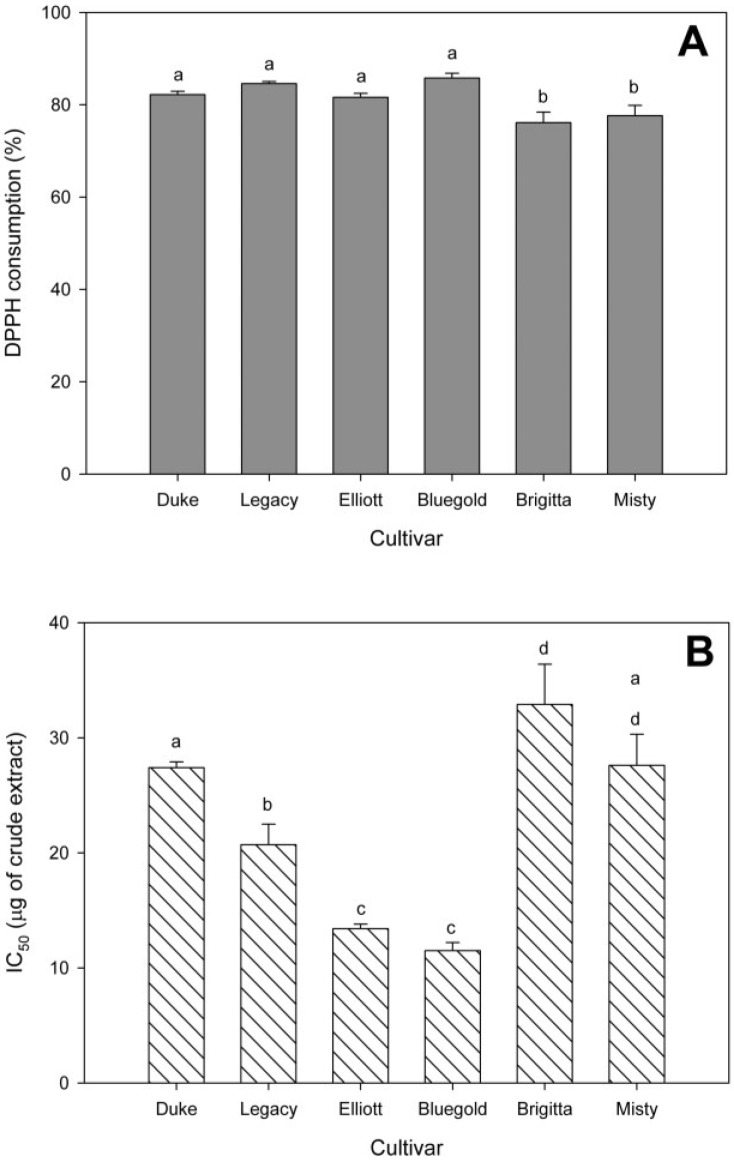
1,1-diphenyl-2-picrylhydrazin (DPPH•) free radical-scavenging activity of six *in vitro* highbush blueberry cultivars. (**A**) Total scavenging activity (as a percentage) from 20 μL of mass normalized hydroethanolic extract; (**B**) IC_50_ index of DPPH• scavenging activity of hydroethanolic extracts. Each bar corresponds at the mean of nine independent measurements ± standard error; letters represent significant differences (*p* < 0.05).

This work reports differences in antioxidant activity and composition between cultivars of blueberry cultured *in vitro*. Ethanolic extracts from *in vitro* blueberry cultivars displayed antioxidant activity (Figure 2A). The results show two groups of plants: those with a high antioxidant capacity (up to 80% DPPH• consumption) such as Duke, Legacy, Elliott and Bluegold, and those with lower antioxidant capacity (lower to 80% DPPH• consumption) such as Brigitta and Misty. The IC_50_ (Figure 2B), shows that the cultivars with better antioxidant activity were Elliott and Bluegold (IC_50_ of 13.4 ± 0.4 and 11.5 ± 0.7 μg, respectively) and with less antioxidant capacity were Duke, Brigitta and Misty (IC_50_, 27.4 ± 0.5, 32.9 ± 3.5 and 27.6 ± 2.7 μg, respectively).

**Figure 3:**
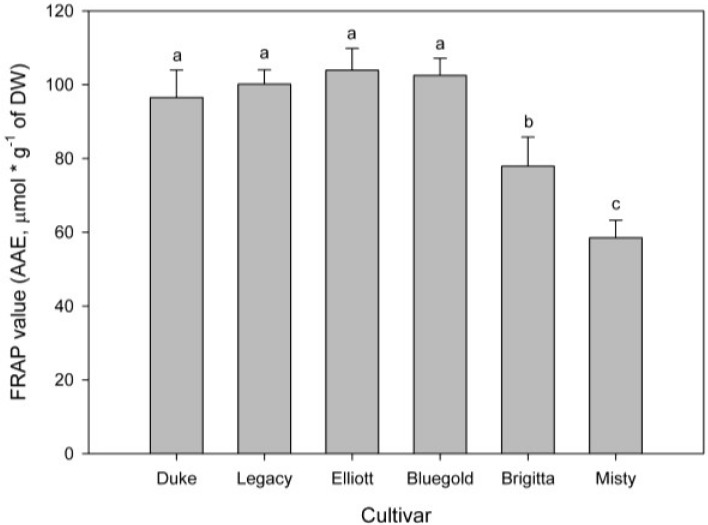
Antioxidant power measured by the Ferric reducing antioxidant power (FRAP) method for six *in vitro* highbush blueberry cultivars. Each bar corresponds at the mean of nine independent measurements ± standard error; letters represent significant differences (*p* < 0.05).

The FRAP assay (Figure 3) show a similar pattern to DPPH• scavenge assay. The FRAP values shows that cultivars Duke, Legacy, Elliott and Bluegold, have a higher reducing power, while Brigitta and Misty cultivars have a lower reducing power (*p* < 0.05). However, a clear difference was observed between the cultivars Brigitta and Misty (*p* < 0.05), which could not be observed in the DPPH• assay. The difference between these two assays may be explained by the methodology. The DPPH• assay considers both synergic and antagonist process and reaches a saturation point, while the FRAP assay does not reach a saturation point and is linear in a wide range. For these reasons both methods are considered complementary [14,15,16].

### 3.3. Total Phenolic Compound Content Determined by Folin-Ciocalteu’s Assay

**Figure 4:**
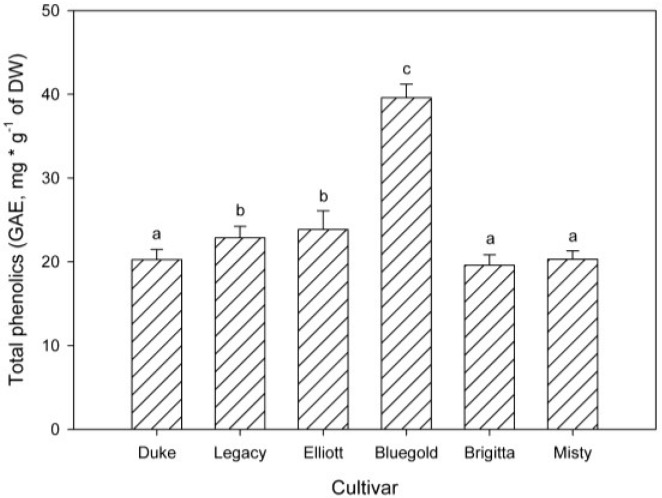
Total phenolic content of six *in vitro* highbush blueberry cultivars. Each bar corresponds at the mean of nine independent measurements ± standard error; letters represent significant differences (*p* < 0.05).

The total content of phenolic compounds present in each cultivar is reported in Figure 4. Cultivar Bluegold contains higher amount of phenolic compounds than the other cultivars (about twice, *p* < 0.05).

### 3.4. Phenolic Profile Determined by HPLC-DAD and LC-MS/MS

**Figure 5:**
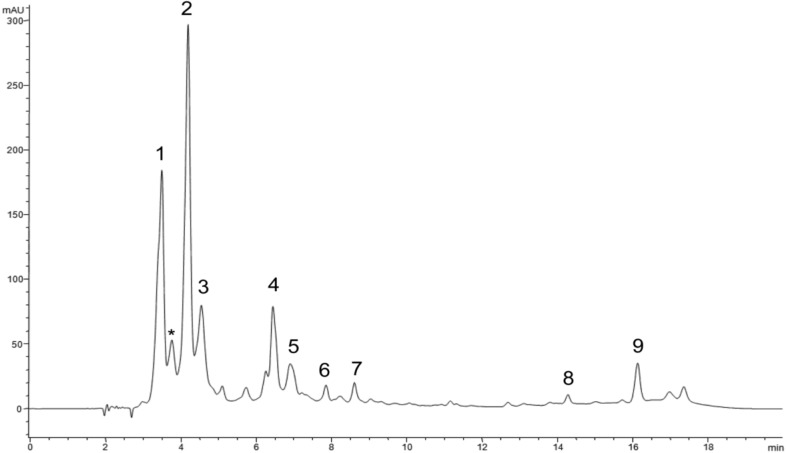
High performance liquid chromatography with diode array detector (HPLC-DAD) chromatogram of hydroethanolic extract of highbush blueberry *cv.* Bluegold. Chromatogram was registered at 280 nm; * background of detector, does not present a UV spectra.

**Figure 6:**
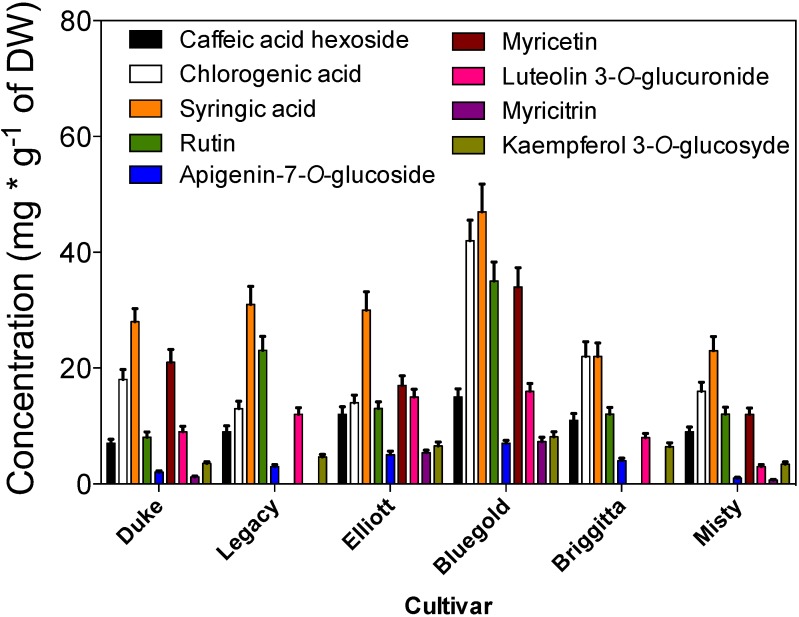
Total concentration of identified compounds in the six *in vitro* highbush blueberry cultivars. Each bar corresponds at the mean of three independent measurements ± standard error (*p* < 0.05).

The phenolic profile of the extracts obtained by HPLC-DAD is shown in Figure 5 and Table 1. By comparing their retention time and UV-Vis spectral properties with those of pure standards, we detected 9 peaks, seven of which were present in all cultivars. The main compounds present in all cultivars were chlorogenic acid, syringic acid and rutin (Figure 5, Table 1). In addition, myricetin (peak #6) and peak #8, was not detected in cultivars Brigitta and Legacy (Figure 6). The ultraviolet spectra and LC-MS/MS analysis corroborates the presence of chlorogenic acid, syringic acid, rutin and myricetin. In addition, peak #1 was identified as caffeic acid hexoside, peak #5 was identified as apigenin-7-*O*-glucoside [17], peaks #7 and #8 were identified luteolin 3-*O*-glucuronide and myricitrin, respectively [18] and finally peak #9 was identified as kaempferol 3-*O*-glycosyde [19].

The IC_50_ of identified compounds compared with IC_50_ of crude extract (Figure 2) and determined by DPPH assay, suggest that the antioxidant activity is mainly due to chlorogenic acid and myricetin (Table 1). Chlorogenic acid is widely distributed in berry crops used as natural antioxidants [20]. Their antioxidant activity is associated to some extent with the number of hydroxyl groups in their molecular structure [21]. Flavonols, such as myricetin, showed high antioxidant activity that correlates with their structure [22].

**Table 1:** Main phenolic compounds identified in blueberry extracts by HPLC and LC-MS/MS. The numbers correspond to peaks in Figure 5 and structures of Figure 7.

Peak Number	Retention Time	UV Spectra	Compound	IC_50_ (μg) *
1	3.5	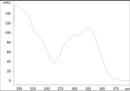	Caffeic acid hexoside	nd
2	4.2	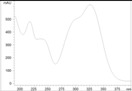	Chlorogenic acid	4.18
3	4.7	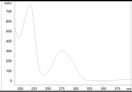	Syringic acid	7.28
4	6.4	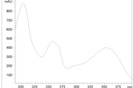	Rutin	9.25
5	6.9	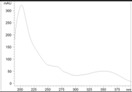	Apigenin-7-*O*-glucoside	nd
6	8.1	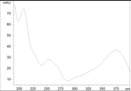	Myricetin	3.20
7	15.8	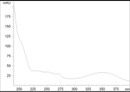	Luteolin 3-*O*-glucuronide	nd
8	16.2	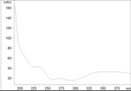	Myricitrin	nd
9	17.4	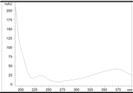	Kempferol 3-*O*-glucosyde	nd

* IC_50_ as calculated using DPPH assay and commercial standards with serial dilutions and lineal regression (*n* = 6), nd = non-determined.

**Figure 7:**
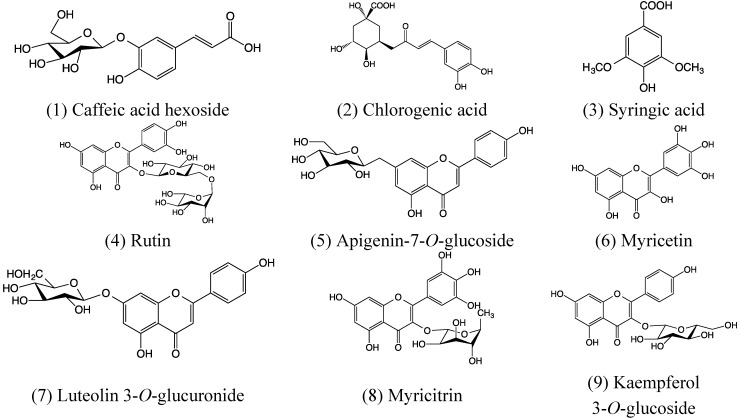
Structures determined for highbush blueberry using UV and MS/MS spectra.

The antioxidant activity of the extracts analyzed was comparable to blueberry fruits (e.g., IC_50_ of Misty fruits is 21.95 μg v/s *in vitro* plants that are 27.6 μg). The very high content of these phytochemicals, especially of chlorogenic acid, syringic acid, rutin and myricetin in extracts of blueberry plants growing *in vitro* conditions, suggest that *in vitro* culture of blueberry is a valuable alternative to produce phenolic compounds with the high demand in the market, especially chlorogenic acid [20].

Plant tissue culture represents an alternative for the production of secondary metabolites with biological activity that are of interest to humans. The obtaining of phytochemicals from the leaves or fruits is affected by environmental factors, such as UV-B radiation, water availability, temperature and diseases [23]. Then, plant organ culture is of greatzhe interest as an alternative for obtaining chemicals from blueberry shoots by reducing the time interval to harvest. Moreover, it allows continuous biomass production. This paper was shown that *in vitro* culture of blueberry is a good alternative to obtain antioxidant molecules such as chlorogenic acid.

The amount (expressed in mg g^−1^ of DW) of each identified compound is reported in Figure 6. In the analyzed extracts from *in vitro* plants, chlorogenic acid was the most abundant compound followed by syringic acid and rutin and myricetin.

## 4. Conclusions

In conclusion, our analyses demonstrate that *in vitro* cultures of blueberries are a good source of antioxidant compounds, especially Bluegold cultivar. Through tissue culture, plant material can be produced continuously to make extracts, whose chemical composition is highly reproducible. Our analytical measurement suggests that the antioxidant activity of the extracts is mainly related to the content of chlorogenic acid, myricetin, syringic acid and rutin. Further analyses are needed to evaluate the cellular toxicity of the *in vitro* shoots extract.
